# Social isolation, loneliness, and subjective wellbeing among Chinese older adults during the COVID-19 pandemic

**DOI:** 10.3389/fpubh.2024.1425575

**Published:** 2024-10-08

**Authors:** Haijun Hao, Mengqi Du, Junyue Yue

**Affiliations:** ^1^Department of Social Welfare, Jeonbuk National University, Jeonju, Republic of Korea; ^2^Department of Media and Communication Studies, Jeonbuk National University, Jeonju, Republic of Korea

**Keywords:** social isolation, loneliness, subjective wellbeing, Chinese older adults, COVID-19 pandemic

## Abstract

The COVID-19 pandemic has brought unprecedented challenges to the wellbeing of the older adults worldwide. Both social isolation and loneliness are associated with decreased subjective wellbeing, but it is uncertain whether their effects are independent or if loneliness represents the affective pathway through which social isolation impairs subjective wellbeing. We therefore assessed the extent to which the association between social isolation and subjective wellbeing is mediated by loneliness. We utilized data from the 2020 China Family Panel Studies (CFPS) and focused on a sample of 3,821 individuals aged 60 and above as the participants for our study. The results revealed a significant negative association between social isolation and subjective wellbeing among the older adults during the COVID-19 pandemic. Furthermore, loneliness was found to mediate this relationship, indicating that social isolation led to increased feelings of loneliness, which in turn negatively impacted subjective wellbeing. These findings highlight the detrimental effects of social isolation and loneliness on the wellbeing of the older adults in China during the pandemic. The implications of these results emphasize the need for interventions and support systems that address social isolation and loneliness among the older adults, promoting their wellbeing and overall mental health during challenging times such as the COVID-19 pandemic.

## 1 Introduction

The outbreak of the COVID-19 pandemic has compelled the Chinese government to implement a series of measures aimed at curtailing the spread of the virus. These measures include the imposition of strict social distancing policies to reduce interactions between people. In January 2020, Wuhan, Hubei Province, for the first time in China, imposed a variety of lockdown measures, including the closure of non-essential businesses and public transportation and restrictions on the movement of individuals (1). This was followed by a city-wide lockdown in many provinces and cities, where residents’ events were cancelled and gatherings were discouraged. However, these policies have posed significant challenges for the older adults in terms of their ability to engage in face-to-face communication with family and friends, leading to profound feelings of loneliness and depression (2, 3). Previous studies have demonstrated that social distancing policies increase loneliness among the older adults (4). Prolonged loneliness may lead to depression and a decrease in subjective wellbeing (5). In addition, in China that the rate of older than 65 years with COVID-19 infection was 15.1%, which was significantly higher than that of the younger group (6), and the risk of death in this age group was reported to be 23 times higher than those under 65 (7). When older people perceive these risks, they can trigger psychological problems such as fear (8). These psychological problems not only affect people’s mental health, but may also affect their life satisfaction and wellbeing (9–11).

Social isolation is recognized as a risk factor for poor health and diminished wellbeing (12–14). A recent report also listed social isolation in late life as one of the key modifiable risk factors for dementia (15). Some argue that the health risks associated with isolation and loneliness are comparable to the well-established detrimental effects of smoking and obesity. In old age, social isolation and loneliness pose significant challenges due to reduced economic and social resources, as well as functional limitations. Furthermore, the COVID-19 pandemic has underscored social isolation as a potential risk factor that may exacerbate psychological distress (16). Previous studies have indicated that the pandemic reduces the subjective wellbeing among the older adults due to their inability to participate in social activities and maintain a normal daily routine as a result of isolation policies (17–19). The older adults are a particularly vulnerable group during the pandemic due to age-related changes and health conditions, making them more susceptible to disease and psychological stress (20). Consequently, social distancing policies during the pandemic have imposed greater challenges and pressures on the health and wellbeing of the older adults.

Loneliness is a prevalent experience among older adults, often attributed to age-related changes and shifts in social roles (21). Extensive literature highlights the significant correlation between loneliness and psychological wellbeing among older adults (5, 22, 23). The implementation of social distancing policies during the COVID-19 pandemic has had profound impacts on the social relationships and activities of older adults, potentially exacerbating their feelings of loneliness (2, 24). Furthermore, this can result in a decrease in the subjective sense of wellbeing among older adults (17). Recent research conducted in China has indicated that more than 20% of older adults frequently experience feelings of loneliness, with females, low-income individuals, and rural residents being particularly vulnerable (25, 26). The social distancing measures and travel restrictions implemented during the pandemic have hindered older adults’ connections with their families and society, intensifying their feelings of loneliness (3). Moreover, the mental health of older adults, especially those facing adverse living conditions, has been negatively affected during the pandemic (27). Research suggests that supporting older adults through social support, hobbies, and positive emotions is a viable strategy for alleviating their feelings of loneliness and improving subjective wellbeing (28–30).

Loneliness is commonly defined by researchers as the subjective perception of feeling socially isolated, while social isolation refers to the objective state of being physically separated from others (31). Both social isolation and loneliness have consistently been linked to negative psychological outcomes, such as depression (32, 33), and a reduction in subjective wellbeing (34–36). An essential scientific inquiry arises regarding the nature of the relationship between social isolation and loneliness, and their respective impacts on subjective wellbeing. It is crucial to determine whether loneliness, with its associated biological factors, acts as a mechanism through which social isolation affects subjective wellbeing, or if social isolation and loneliness are distinct processes that independently contribute to subjective wellbeing. Resolving this question is of great significance as it can help identify effective strategies for intervention and support tailored to the needs of older individuals. Therefore, the aim of our study was to investigate the associations between social isolation, loneliness, and subjective wellbeing in a nationally representative sample of older adults, while also examining the extent to which loneliness mediates the relationship between social isolation and subjective wellbeing.

Subjective wellbeing refers to an individual’s overall evaluation of their life based on personal criteria, providing a comprehensive and relatively stable perspective that serves as a psychological indicator of life quality (37). It encompasses a holistic subjective assessment that includes the pleasurable experiences of the body and mind, emotional wellbeing, and life satisfaction. In this study, subjective wellbeing was assessed using two indicators: life satisfaction and personal wellbeing, which takes into account considerations of future security, personal relationships, and the impact of positive events (38). The conceptualization of subjective wellbeing by Diener et al. (38) highlights the significance of including life satisfaction as a key component. Additionally, Cummins et al. (39) proposed the Theory of Subjective Wellbeing Homeostasis, which suggests that subjective wellbeing comprises various dimensions, such as satisfaction with the standard of living, future security, personal relationships, the impact of positive or negative events, and health. Therefore, this study measured subjective wellbeing through assessments of life satisfaction, confidence in the future, interpersonal relationships, and experiences of happiness (40). Given the outbreak of the COVID-19 pandemic, it is expected that older adults would face increased social isolation and loneliness. Consequently, we hypothesized that heightened social isolation and loneliness would be associated with reduced subjective wellbeing during the COVID-19 pandemic.

Hence, the aim of this study is to examine the impact of social isolation during the pandemic on the subjective wellbeing of older adults. Furthermore, we will examine the mediating effect of loneliness on the association between social isolation and subjective wellbeing. This investigation aims to offer valuable insights into the underlying mechanisms that drive the relationship between social isolation, loneliness and subjective wellbeing in older adults during the COVID-19 pandemic. Ultimately, the findings of this study aim to inform the development of targeted interventions to promote wellbeing among old adults.

## 2 Materials and methods

### 2.1 Data and study population

We utilized data from the China Family Panel Studies (CFPS), a comprehensive nationwide social survey jointly conducted by the Institute of Social Science Survey at Peking University and the Survey Research Center at the University of Michigan (41). The sample of CFPS covered 25 provinces (excluding Hong Kong, Macao, Taiwan, Xinjiang, Tibet, Qinghai, Inner Mongolia, Ningxia, and Hainan), representing 94.5% of the total population in Mainland China. Our analysis focused on the CFPS data from 2020, which were the most recently available. The survey was conducted from July 2020 to the end of that year, when the epidemic in China had been effectively prevented and controlled, with fewer new cases and the prevention and control of the epidemic entering a normalized phase (42). At the same time, in order to avoid the increased risk of epidemic transmission, the Chinese government has urged residents to reduce unnecessary outings and avoid large gatherings. Therefore, the 2020 Survey mainly used telephone interviews. If the respondent requests a face-to-face interview, the interviewer will conduct the face-to-face interview according to local public health policy requirements. The study received approval from the Peking University Biomedical Ethics Committee (IRB00001052-14010), and we included participants aged 60 years and above in our sample. Out of the initial 28,590 survey participants in 2020, individuals below the age of 60 (*n* = 14,252) and those with incomplete information (*n* = 10,516) were excluded, resulting in a final analytical sample of 3,821 eligible participants.

### 2.2 Measurements

#### 2.2.1 Subjective wellbeing

We assessed the participants’ subjective wellbeing, encompassing aspects such as life satisfaction, future confidence, interpersonal relationships, and experience of happiness. To measure these constructs, we selected four items: “satisfaction with your life” (rated on a scale of 1–5), “confidence in your future” (rated on a scale of 1–5), “the quality of your relationships” (rated on a scale of 0–10), and “how happy are you” (rated on a scale of 0–10). To ensure a more accurate reflection of subjective wellbeing, we adjusted the scores by transforming 0 points into 1 point for “the quality of your relationships” and “how happy are you,” and applied a two-fold weighting to the scores of “satisfaction with your life” and “confidence in your future” (40).

#### 2.2.2 Social isolation

The index of social isolation was generated by assigning one point if participants were not married (never married, separated, divorced, widowed), living alone, having less than weekly contact (including face-to-face, telephone, or e-mail) with their children, living in the rural rather than an urban area, and not using internet (14, 43). Scores ranged from 0 to 5, with higher scores indicating greater social isolation. Given the constraints on social activities imposed by social distancing policies during the pandemic, this study incorporates internet usage, which is less affected by these policies, as a measure of social isolation (44).

#### 2.2.3 Loneliness

In this study, we examine the construct of loneliness as the mediated variable. Loneliness is measured by assessing participants’ self-reported frequency of experiencing loneliness within a week. To operationalize this, we employ a 4-point ordinal scale response option, ranging from 1 (less than a day) to 4 (5 days or more). Higher scores on this scale indicate higher levels of loneliness (45).

#### 2.2.4 Covariates

In our study, we included several covariates that have been identified as having associations with subjective wellbeing among older adults. These covariates encompassed gender, age, educational level, socioeconomic status (SES), pension, chronic disease and subjective health (40, 46). SES was assessed using two items: “What is your personal income?” (SES-economy) and “What is your social status?” (SES-society). Respondents rated these items on a 5-point scale, ranging from 1 (very low) to 5 (very high).

### 2.3 Data analysis

**Figure 1:**
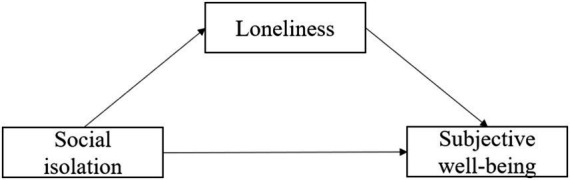
Research model.

The primary objective of this study is to explore the influence of social isolation on subjective wellbeing among the older adults in China during the COVID-19 pandemic. Moreover, we seek to examine whether loneliness mediates the relationship between social isolation and subjective wellbeing. The statistical analysis was conducted using STATA 17 software, employing regression models to examine the associations between social isolation, loneliness, and subjective wellbeing. Additionally, pathway analysis was utilized to assess the mediating role of loneliness (Figure 1).

## 3 Results

### 3.1 Characteristics of the study population

Table 1 displays the demographic characteristics of the study sample. The sample comprised 2,024 males (52.97%) and 1,797 females (47.03%), with an average age of 67.88 years. The age range of participants in this study was 60–93 years old. Education levels varied, with the majority of participants reporting no formal education (44.18%), followed by primary education (23.13%), junior high school education (19.79%), and high school education or above (12.90%). A significant proportion of participants reported receiving a pension (68.86%). Furthermore, a considerable number of participants reported having a chronic disease (68.86%). The average economic status was 3.21 (range 1–5), and the average social status was 3.52 (range 1–5). The findings indicate that the majority of individuals perceived their socioeconomic status to be above average. Participants had an average subjective health rating of 2.66 (range 1–5). The results suggest that a significant proportion of older adults perceive their health status to be within the average range. Regarding the descriptive statistics, the mean score for subjective wellbeing is 8.20, indicating a relatively high level of wellbeing on average. Social isolation has a mean score of 2.42, indicating a moderate level of social isolation. Loneliness has a mean score of 1.52, suggesting a relatively low level of loneliness on average.

**Table 1:** Characteristics of the study population (*N* = 3,821).

Variables		Frequency (*N*)	Percent (%)
Gender	Male	2,024	52.97
	Female	1,797	47.03
Age (Mean/SD) (Range)		67.88(SD 5.67) (60 ~ 93)	
Education	Unschooled	1,688	44.18
	Primary	884	23.13
	Junior	756	19.79
	High School and above	493	12.90
Economic status (Mean/SD)		3.21 (SD 1.17)	
Social status (Mean/SD)		3.52 (SD 1.11)	
Pension	No	1,190	31.14
	Yes	2,631	68.86
Chronic disease	No	1,190	31.14
	Yes	2,631	68.86
Social isolation (Mean/SD) (Range)		2.42 (SD 1.02) (0–5)	
Loneliness (Mean/SD) (Range)		1.52 (SD 0.86) (1–4)	
Subjective health (Mean/SD) (Range)		2.66 (SD 1.28) (1.5–10)	

### 3.2 Correlation of the main variables

Table 2 presents the correlation coefficients and descriptive statistics for subjective wellbeing, social isolation, and loneliness. In terms of correlations, subjective wellbeing demonstrates a weak negative correlation with social isolation −0.084 (*p* < 0.001) and a moderate negative correlation with loneliness −0.191 (*p* < 0.001). These findings suggest that higher levels of social isolation and loneliness are associated with lower subjective wellbeing. In addition, the correlation coefficient of 0.196 (*p* < 0.001) between social isolation and loneliness indicates a positive relationship between social isolation and loneliness. This suggests that further analysis can be conducted.

**Table 2:** Correlation and characteristics of the main variables.

	Subjective wellbeing	Social isolation	Loneliness
Subjective wellbeing	1.000		
Social isolation	−0.084***	1.000	
Loneliness	−0.191***	0.196***	1.000

***p < 0.001.

### 3.3 Mediation model

Table 3 showed the model fit indices for the collected values and the recommended values among older adult model. In terms of model fit, the χ^2^ value is 105.895 (*p* < 0.001), indicating a significant discrepancy between the observed and expected covariance matrices. The root mean square error of approximation (RMSEA) is 0.050, which falls below the recommended threshold of 0.08, suggesting an acceptable fit. The standardized root mean square residual (SRMR) is 0.021, below the recommended threshold of 0.05, indicating a good fit. The comparative fit index (CFI) is 0.927, which is above the recommended threshold of 0.9, indicating a satisfactory fit. Furthermore, the *p*-value for the probability close (pclose) statistic is 0.473, which is above the recommended threshold of 0.05, indicating a good fit between the observed data and the hypothesized model. Overall, based on the recommended values for the model fit indices, the collected values indicate a reasonably good fit between the proposed model and the observed data.

**Table 3:** Model fit among older adult model.

	*χ* ^2^	RMSEA	SRMR	CFI	pclose
Collected values	105.895***	0.050	0.021	0.927	0.473
Recommended values	–	<0.08	<0.05	>0.90	>0.05

*p < 0.05, **p < 0.01, ***p < 0.001.

**Figure 2:**
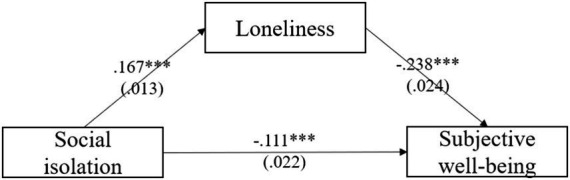
Mediating effect of loneliness among older adults.

Table 4 showed the mediation model among older adults, which revealed a mediation role of loneliness in the relationship between social isolation and subjective wellbeing. Model controlled for gender, age, education, economic status, social status, pension, chronic disease and subjective health. The mediating effects of loneliness were − 0.040 (*p* < 0.001), and the mediating effects accounted for 26.5% of the total effects. Both the direct effects and the indirect effects were significant. Figure 2 depicts the mediation model of loneliness between social isolation and subjective wellbeing. In our investigation of the influence of social isolation and loneliness on subjective wellbeing, we observed that loneliness (*β* = −0.238, *p* < 0.001) exerted a greater impact on subjective wellbeing when compared to social isolation (*β* = −0.111, *p* < 0.001).

**Table 4:** Subjective wellbeing with social isolation mediated via loneliness among older adults.

Effects	*β*	SE	*p*	95%CI	
				Lower	Upper
Model 1 (Adjusted for covariates)					
Direct effects	−0.111	0.022	0.000	−0.155	−0.067
Indirect effects	−0.040	0.005	0.000	−0.050	−0.030
Total effects	−0.151	0.022	0.000	−0.194	−0.107

*p < 0.05, **p < 0.01, ***p < 0.001. Covariates including gender, age, education, economic status, social status, pension, chronic disease and subjective health.

#### 3.3.1 Comparative analysis of middle-aged and older adults

In addition to the primary analysis focused on older adults aged 60 and above, a comparative analysis was conducted to examine the impact of social isolation and loneliness on cognitive function among middle-aged adults. We selected 7,252 middle-aged individuals, aged 40–60 years, from the CFPS dataset as the subjects for comparative analysis. The same path analysis model was applied to both age cohorts, allowing for a direct comparison of the effects across these two age groups.

Table 5 showed the model fit indices for the collected values and the recommended values among middle-aged adult model. In terms of model fit, the χ^2^ value is 98.798 (*p* < 0.001), indicating a significant discrepancy between the observed and expected covariance matrices. The root mean square error of approximation (RMSEA) is 0.037, which falls below the recommended threshold of 0.08, suggesting an acceptable fit. The standardized root mean square residual (SRMR) is 0.015, below the recommended threshold of 0.05, indicating a good fit. The comparative fit index (CFI) is 0.962, which is above the recommended threshold of 0.90, indicating a satisfactory fit. Furthermore, the *p*-value for the probability close (pclose) statistic is 0.557, which is above the recommended threshold of 0.05, indicating a good fit between the observed data and the hypothesized model. Overall, based on the recommended values for the model fit indices, the collected values indicate a reasonably good fit between the proposed model and the observed data.

**Table 5:** Model fit among middle-aged adult model.

	*χ* ^2^	RMSEA	SRMR	CFI	pclose
Collected values	98.798***	0.037	0.015	0.962	0.577
Recommended values	–	<0.08	<0.05	>0.90	>0.05

*p < 0.05, **p < 0.01, ***p < 0.001.

**Figure 3:**
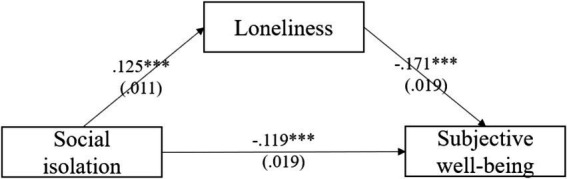
Mediating effect of loneliness among middle-aged adults.

Table 6 showed the mediation model among middle-aged adults, which revealed a mediation role of loneliness in the relationship between social isolation and subjective wellbeing. Model controlled for gender, age, education, economic status, social status, pension, chronic disease and subjective health. The mediating effects of loneliness among middle-aged adults were −0.021 (*p* < 0.001), and the mediating effects accounted for 15.0% of the total effects. Both the direct effects and the indirect effects were significant. Figure 3 depicts the mediation model of loneliness between social isolation and subjective wellbeing. The comparative analysis results between Table 4 (older adults) and Table 6 (middle-aged adults) indicate that the effects of social isolation and loneliness are also significant in the middle-aged group, but they are more pronounced in the older adults. Specifically, the total effect of social isolation and loneliness in the mediation analysis is greater in the older adults’ model (−0.151) compared to the middle-aged model (−0.140). Additionally, the proportion of the mediation effect in the older adults model (26.5%) is also larger than that in the middle-aged model (15%).

**Table 6:** Subjective wellbeing with social isolation mediated via loneliness among middle-aged adults.

Effects	*β*	SE	*p*	95%CI	
				Lower	Upper
Model 1 (Adjusted for covariates)					
Direct effects	−0.119	0.020	0.000	−0.159	−0.080
Indirect effects	−0.021	0.005	0.000	−0.056	−0.035
Total effects	−0.140	0.022	0.000	−0.201	−0.116

Table 7 presents the specific results of the comparative analysis between middle-aged and older adults. In the older adults’ Model 1, we regressed subjective wellbeing onto social isolation without including loneliness. In Model 3, we incorporated the effect of loneliness, resulting in a decrease in the *β* value from −0.141 to −0.111, a reduction of 0.030. Similarly, in the middle-aged adults’ Model 4, we regressed subjective wellbeing onto social isolation without including loneliness, and in Model 6, we added loneliness. This inclusion led to a decrease in the β value from −0.145 to −0.119, a reduction of 0.026. Using a three-step method, the change in β values further substantiates the mediating role of loneliness in both middle-aged and older adult groups.

**Table 7:** Comparative analysis results between middle-aged and older adults.

	Older adults			Middle-aged adults		
	Model 1 (xy)	Model 2 (xm)	Model 3 (xmy)	Model 4 (xy)	Model 5 (xm)	Model 6 (xmy)
	*β*	*β*	*β*	*β*	*β*	*β*
Social isolation	−0.141***	0.167***	−0.111***	−0.145***	0.125	−0.119***
Loneliness			−0.238***			−0.171***
Constant	5.253***	1.49***	5.61***	5.283***		5.97***
adj *R*^2^	0.226	0.060	0.244	0.239	0.052	0.275
*F*	124.74***	29.56	124.51***	254.17***	43.81***	276.15***

*p < 0.05, **p < 0.01, ***p < 0.001. Covariates including gender, age, education, economic status, social status, pension, chronic disease and subjective health.

## 4 Discussion

This study aimed to examine the impact of social isolation on subjective wellbeing among the older adults in China during the COVID-19 pandemic, with a focus on the mediating role of loneliness. Although social isolation and loneliness are detrimental to subjective wellbeing across the mid to late adulthood, older adults are particularly vulnerable. The findings shed light on the psychological consequences of social isolation and loneliness during a period of crisis and provide insights into potential interventions to improve the wellbeing of older adults in similar circumstances.

The findings of our study validate and expand upon previous research, which demonstrates a significant negative association between social isolation and subjective wellbeing among the older adults in China during the COVID-19 pandemic. These results are consistent with existing literature, including studies by Chappell & Badger (47) and Clair et al. (48), which consistently highlight the detrimental effects of social isolation on mental health and wellbeing, particularly in older adults. The implementation of strict physical distancing measures and restrictions on social interactions during the pandemic has greatly limited opportunities for social engagement and support, resulting in increased levels of loneliness and subsequent declines in subjective wellbeing among the older adults. Moreover, our study contributes to the understanding of the underlying mechanisms linking social isolation and subjective wellbeing by identifying loneliness as a significant mediating factor. Extensive research, such as the work of Okruszek et al. (49) and Lorber et al. (50), consistently links loneliness to various negative mental health outcomes, including an elevated risk of depression, anxiety, and diminished life satisfaction. The COVID-19 pandemic has intensified feelings of loneliness among the older adults due to the disruption of their social networks, limited access to social activities, and heightened fear of infection. In addition, the database was collected from a relatively healthy population, especially vulnerable subgroups, such as the very older adult and patients with chronic or severe diseases, who may experience more feelings of loneliness and distress when deprived of personal social contacts (51). Our findings highlight the critical importance of addressing loneliness as a key factor in promoting subjective wellbeing among the older adults during periods of social isolation.

The identification of loneliness as a mediator suggests potential pathways through which social isolation impacts subjective wellbeing. The absence of social interaction and meaningful connections may contribute to heightened feelings of loneliness, which, in turn, can lead to a decline in subjective wellbeing. Therefore, interventions aimed at mitigating the negative effects of social isolation should prioritize addressing loneliness as a primary outcome. To achieve this, virtual social support programs, online group activities, and outreach initiatives can be implemented to maintain regular communication and foster social connections with the older adults. These interventions can help alleviate the sense of isolation and provide avenues for social interaction, ultimately promoting subjective wellbeing. Additionally, it is crucial to allocate sufficient resources and support to address the mental health implications of social isolation and loneliness among the older adults, ensuring that appropriate mental health services are accessible and readily available.

It is important to note that our study focused specifically on the older adults in China during the COVID-19 pandemic, and the findings may not be generalizable to other populations or contexts. Cultural factors, social norms, and individual differences may influence the experience of social isolation, loneliness, and subjective wellbeing. Future research should aim to replicate these findings in diverse populations and explore potential cultural variations in the impact of social isolation and loneliness on subjective wellbeing. Despite these limitations, our study contributes to the growing body of literature on the psychological impact of social isolation and loneliness, particularly during times of crisis such as the COVID-19 pandemic. By highlighting the mediating role of loneliness, our findings underscore the importance of addressing social isolation and promoting social connectedness to enhance the wellbeing of the older adults. Policymakers, healthcare providers, and community organizations should consider implementing targeted interventions to alleviate loneliness and improve the subjective wellbeing of older adults, not only during times of crisis but also in normal circumstances.

In conclusion, our study not only reinforces the existing body of knowledge regarding the detrimental impact of social isolation on subjective wellbeing among the older adults during the COVID-19 pandemic but also contributes by identifying loneliness as a significant mediator in this relationship. Our findings underscore the importance of implementing interventions that specifically target loneliness to enhance the wellbeing of the older adults. By offering virtual social support programs, facilitating online group activities, and establishing outreach initiatives, societies can maintain regular communication with older individuals, promoting social connectedness and improving their overall wellbeing. Additionally, it is crucial to provide adequate mental health support and resources to address the psychological consequences of social isolation and loneliness. These efforts are not only essential during times of crisis but also in normal circumstances, ensuring the long-term wellbeing of the older adults.

## 5 Conclusion

In conclusion, this study sheds light on the impact of social isolation on the subjective wellbeing of the older adults in China during the COVID-19 pandemic, with a particular focus on the mediating role of loneliness. Our findings highlight the detrimental effects of social isolation on subjective wellbeing, as evidenced by lower levels of life satisfaction, decreased emotional wellbeing, and diminished overall happiness among older adults. Importantly, we found that loneliness partially mediates the relationship between social isolation and subjective wellbeing, suggesting that the emotional experience of loneliness plays a significant role in the negative psychological outcomes associated with social isolation. These results underscore the importance of addressing both social isolation and loneliness in interventions and support systems targeted towards older individuals, particularly during times of crisis such as the COVID-19 pandemic. By implementing strategies to mitigate social isolation and foster social connections, healthcare professionals, policymakers, and communities can contribute to the enhancement of subjective wellbeing and overall quality of life among the older adults. Further research is warranted to explore additional factors and interventions that can effectively alleviate social isolation and loneliness, thereby promoting the wellbeing and mental health of older adults in challenging times.

## Data Availability

Publicly available datasets were analyzed in this study. This data can be found here: we used a publically available dataset, China Family Panel Studies (CFPS; https://www.isss.pku.edu.cn/cfps/en/).
